# Influence of Intraoperative Nociception during Hip or Knee Arthroplasty with Supplementary Regional Anaesthesia on Postoperative Pain and Opioid Consumption

**DOI:** 10.3390/medicina59061166

**Published:** 2023-06-17

**Authors:** Claudia Neumann, Lena Gehlen, Leonie Weinhold, Nadine Straßberger-Nerschbach, Martin Soehle, Evgeniya Kornilov, Marcus Thudium

**Affiliations:** 1 Department of Anaesthesiology and Intensive Care Medicine, University Hospital Bonn, Venusberg Campus 1, 53127 Bonn, Germanymartin.soehle@ukbonn.de (M.S.);; 2 Department of Medical Biometry, Informatics and Epidemiology, University of Bonn, Venusberg Campus 1, 53127 Bonn, Germany; 3 Department of Anaesthesia, Rabin Medical Center, Beilinson Hospital, 39 Jabotinsky Street, Petach Tikva 4941492, Israel; 4 Department of Neurobiology, Weizmann Institute of Science, 234 Herzl Street, Rehovot 7610001, Israel

**Keywords:** nociception, joint surgery, regional anaesthesia

## Abstract

*Background and Objectives*: Early postoperative mobilization is central for postoperative outcomes after lower extremity joint replacement surgery. By providing adequate pain control, regional anaesthesia plays an important role for postoperative mobilization. It was the objective of this study to investigate the use of the nociception level index (NOL) to determine the effect of regional anaesthesia in hip or knee arthroplasty patients undergoing general anaesthesia with additional peripheral nerve block. *Materials and Methods*: Patients received general anaesthesia, and continuous NOL monitoring was established before anaesthesia induction. Depending on the type of surgery, regional anaesthesia was performed with a Fascia Iliaca Block or an Adductor Canal Block. *Results*: For the final analysis, 35 patients remained, 18 with hip and 17 with knee arthroplasty. We found no significant difference in postoperative pain between hip or knee arthroplasty groups. NOL increase at the time of skin incision was the only parameter associated with postoperative pain measured using a numerical rating scale (NRS > 3) after 24 h in movement (−12.3 vs. +119%, *p* = 0.005). There was no association with intraoperative NOL values and postoperative opioid consumption, nor was there an association between secondary parameters (bispectral index, heart rate) and postoperative pain levels. *Conclusions*: Intraoperative NOL changes may indicate regional anaesthesia effectiveness and could be associated with postoperative pain levels. This remains to be confirmed in a larger study.

## 1. Introduction

Orthopaedic joint arthroplasties can be stressful procedures for patients due to high pain levels, especially in the context of postoperative mobilization [1]. Therefore, regional anaesthesia procedures and pain pumps are regularly used to optimize the intra- and postoperative process.

Unlike processed electroencephalography (EEG) for the assessment depth of anaesthesia, the objective measurement of intraoperative pain and stress has not yet become part of the anaesthesiological standard, although corresponding devices have been available on the market for years [2]. Thus, analgesia in the perioperative setting is usually left to the experience and judgment of the anaesthesiologist.

Since both over- and underdosing of analgesia can lead to undesirable side effects for the patient, individualized administration of pain medication is desirable [3]. So far, the assessment of the effectiveness of a preoperatively established regional anaesthesia is still based on indirect parameters, such as heart rate or blood pressure changes after certain stimuli, with consequent adjustment of the analgesia.

With the use of supplemental regional anaesthesia, it is of importance to have early feedback on block effectiveness to be able to provide adequate analgesia. Regional anaesthesia is also an integral part of multimodal pain management within the framework of ERAS (Enhanced Recovery After Surgery) protocols. Here, however, the dosage of analgesics is commonly based on a fixed standard and is not individually tailored to patient requirements [4].

The application of a monitor for the objectifiable recording of pain and stress under anaesthesia could be advantageous to the administration of analgesics at the end of anaesthesia at an optimal dosage. With this study, we aimed to investigate whether intraoperative nociception is associated with postoperative pain or analgesic consumption using the nociception level index (NOL) and postoperative and pain scores with the numeric rating scale (NRS). We hypothesized that intraoperative nociception measurement can determine the effectiveness of peripheral nerve blocks in terms of postoperative pain and opioid consumption in patients undergoing lower extremity joint arthroplasty. We hypothesized a relationship between NOL changes at skin incision and during surgery and postoperative pain as well as opioid consumption.

Additionally, in a sub-analysis, it was evaluated how changes in heart rate and bispectral index (BIS) as standard references behave after a stimulus and in relationship to postoperative pain.

## 2. Materials and Methods

In a prospective, monocentric observational study, 45 patients who underwent knee or hip joint replacement in the Department of Orthopaedics and Trauma Surgery at the University Hospital Bonn, and for whom a regional anaesthesia procedure was established preoperatively, were included during the period 01-05/2019.

The study was approved by the University of Bonn ethics committee (Chair: Prof. K. Racké, No 385/17). After informed consent, patients were enrolled in the study. For anaesthesia induction, patients received standard monitoring with electrocardiography (ECG), peripheral oxygen saturation, and non-invasive blood pressure monitoring. Additionally, bispectral index (BIS) monitoring was established, and the finger clip of the PMD-200 NOL monitor (Medasense Biometrics, Ramat Gan, Israel) was attached to the patient for continuous monitoring. Anaesthesia was induced with 0.2–0.3 mg of Fentanyl and 2 mg/kg bodyweight Propofol. Neuromuscular block was achieved with 30–50 mg Rocuronium. Patients were endotracheally intubated and mechanically ventilated. Depending on the surgical procedure, a fascia iliaca block or an adductor canal block as a single-shot procedure was applied. Patients with hip arthroplasty received a fascia iliaca block: using a linear ultrasound transducer placed on the inguinal crease, the femoral artery was located. The iliacus muscle was identified laterally to the artery and medially in relation to the sartorius muscle. Thus, 30–40 mL of Ropivacain 0.375% was injected underneath the iliacus fascia in an in-plane technique. Patients undergoing knee arthroplasty received an adductor canal block. The sartorius muscle was located at mid-thigh level using ultrasound from an antero-medial direction, with the artery, vein, and nerve on the dorso-lateral side. We followed the artery distally with ultrasound to the point where it moved away dorsally into the adductor canal. A volume of 20 mL of Ropivacaine 0.375% was injected via an in-plane technique into the fascial structure on the lateral side of the artery.

During surgery, anaesthesia was continued with a balanced anaesthesia regime consisting of Isoflurane and Remifentanil. For postoperative analgesia, pain pumps were used starting in the post-anaesthesia care unit (PACU), with a mixture of Metamizole and Tramadol in knee replacements and Piritramide in hip replacements, respectively.

All perioperative vital signs and administered medications were recorded and evaluated in the electronic patient management data system (PDMS). In addition to the standard monitoring, which included ECG, blood pressure measurement, oxygen saturation, and BIS monitoring, the intraoperative nociception level (NOL) was recorded with a monitor from Medasense (PMD 200), which was blinded for the anaesthesiologist.

Pain levels were assessed in PACU, 24 and 48 h after surgery using the numerical rating scale (NRS). Pain was assessed based on an interview of the patients’ current pain experience at rest and in movement. Assessment and documentation were performed by trained and specialized nursing staff. The opioid consumption documented from the pain pumps was evaluated and converted into morphine equivalents. The NOL display was covered during the procedure to avoid influencing the anaesthesiologist’s decision in the use of analgesic medication.

Primary outcome parameters were the association between NOL values at skin incision as well as during surgery and postoperative pain at 24 and 48 h after surgery. Secondary outcome parameters were:

- Relationship between NOL values at skin incision as well as during surgery and postoperative opioid consumption at 24 and 48 h after surgery.
- Relationship between change in secondary parameters (BIS, HR) at skin incision and postoperative pain at 24 and 48 h after surgery.

### 2.1. Statistical Methods

Baseline characteristics of the study population are presented as means with standard deviations (SDs) or median with interquartile range (IQR) for continuous variables and counts with percentages for categorical variables.

The intraoperative NOL, BIS, and heart rate (HR) values were summarized into the following variables: median during surgery, median value during 60 s before intubation or skin incision, median value during 60 s after intubation or skin incision, and knife-to-skin reaction: percentage change of NOL, BIS, or HR values after skin incision (pre vs. post (%)). Additionally, time-weighted averages (TWA) of NOL values lower than 10 or higher than 25, 30, 35, 40, and 45 were calculated in accordance with the manufacturer’s recommended intraoperative range of 10–25.

Concerning postoperative pain, patients were split into two groups according to numerical rating scale (NRS) values: low pain (NRS ≤ 3) and high pain (NRS > 3). NOL, BIS, and HR variables were tested for differences regarding these two groups using the Wilcoxon Mann–Whitney tests. The association between NOL values and morphine equivalents was investigated using linear regression models.

*p*-values < 0.05 (two sided) were considered statistically significant.

### 2.2. Sample Size Calculation

Assuming equal group size of patients with low and high postoperative pain (NRS ≤ 3 vs. NRS > 3), mean NOL values of 20 and 30 were assumed for the groups, respectively, with a standard deviation of 10. Using the Wilcoxon Mann–Whitney test, a power of 80%, and a level of significance of 5% (two-sided), the resulting sample size is 18 participants per group. Assuming a drop-out rate of 20% due to technical issues, we aimed for a final sample size of 23 patients per group.

## 3. Results

**Figure 1:**
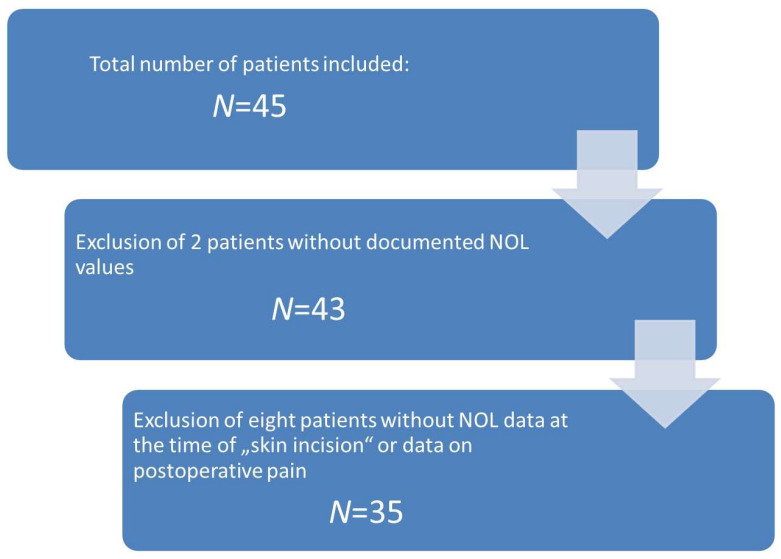
Flowchart of patient inclusion.

Initially, 45 patients were included; after checking for dropout criteria, 35 patients remained for final analysis (3 patients with missing NOL values, 6 patients with no regularly documented incision–suture time). See Figure 1 for patient inclusion.

Of the remaining patients, 18 had received hip arthroplasty and 17 knee arthroplasty. Demographic and surgery related data can be seen in Table 1.

**Figure 2:**
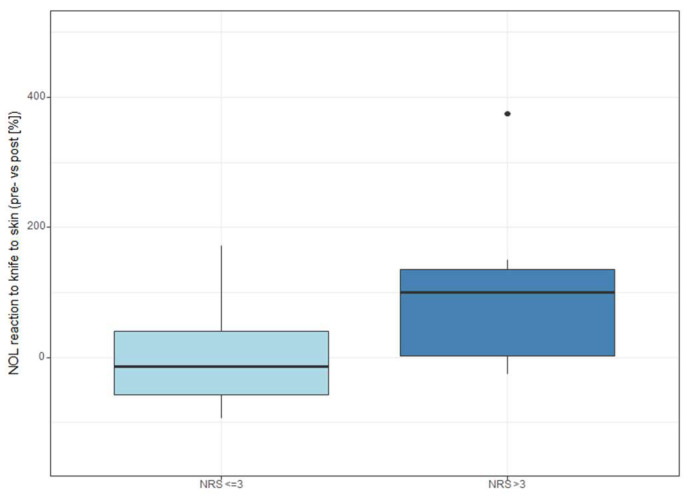
Changes in NOL after skin incision according to postoperative pain on the first postoperative day in movement. NOL: Nociception level index, NRS: Numeric Rating Scale (0–10).

**Figure 3:**
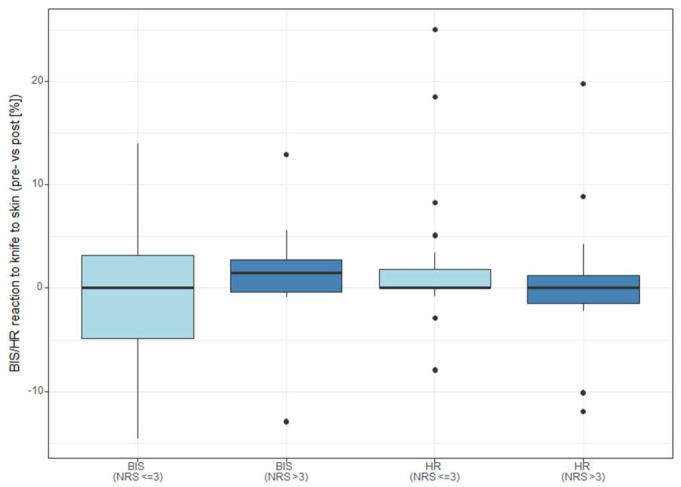
Changes in HR and BIS after skin incision according to postoperative pain on the first postoperative day in movement. BIS: bispectral index, HR: heart rate, NRS: Numeric Rating Scale (0–10).

We found a relationship between the NOL reaction to skin incision and postoperative NRS > 3 in movement within 24 h after surgery (*p* = 0.005). Although a tendency could be observed, this effect was not significant on the second postoperative day (*p* = 0.072). See Figure 2 for NOL reaction to skin incision and postoperative pain. Only two patients had NRS > 3 at rest on the first postoperative day and one patient at rest on the second. None of the absolute NOL values were associated with pain outcomes. There was also no significant association between BIS changes and postoperative NRS groups, nor was this the case for heart rate changes, as shown in Figure 3. Time-weighted averages yielded no substantial results, although a tendency could be observed for TWA < 10 (*p* = 0.055). NOL- and NRS-related data for 24 h after surgery in movement are shown in Table 2 and for 48 h after surgery in Table S1. There was no significant difference in postoperative pain between the two types of surgery (hip and knee arthroplasty). We found no significant association between absolute NOL values or NOL changes and postoperative opioid consumption, as can be seen in Figure S1.

## 4. Discussion

Knee and hip replacements are among the most common joint operations in the field of orthopaedics [5,6,7]. This results in considerable burden on the health system. Data extrapolations have shown that German statutory health insurance spent approximately EUR 1.4 to 1.6 billion per year on hospital treatments for hip arthroplasty between 2003 and 2009. With regard to knee arthroplasty, expenditure for the same period was estimated at EUR 1.0 to 1.3 billion per year [8,9,10].

Early mobilization of patients plays a decisive role, as it has been shown to reduce the length of hospital stay and, thus, save costs [11,12]. However, early mobilization is only possible if adequate pain control is provided. In this context, regional anaesthesia can represent a central element when embedded in multimodal opioid-sparing or opioid-free concepts. Regional anaesthesia after joint arthroplasty has been shown to reduce postoperative pain as well as opioid consumption and side effects, reduce the length of hospital stay, and improve postoperative rehabilitation [13]. Therefore, regional anaesthesia is an integral part of ERAS (Enhanced Recovery After Surgery) protocols, which always follow a multimodal approach [14,15]. In protocols aiming at early mobilization, the goal is to provide improved pain therapy while, at the same time, avoiding opioids because of their side effects. Therefore, there is a need for effective regional anaesthesia, which should ideally be evaluated as early in the process as possible.

Currently, there is no established means of evaluating regional anaesthesia effectiveness during surgery. Therefore, intraoperative nociception measurement may be beneficial in predicting the effectiveness of peripheral nerve blocks and, thus, optimize patient’s postoperative analgesia, also with regard to early mobilization. However, in our patients, elevated nociception levels during anaesthesia do not appear to be associated with postoperative opioid consumption. It has been suggested previously that nociception, as experienced under anaesthesia, is by no means comparable to pain experienced in an awake state [16]. In contrast, we found that patients who reacted less to the stimulus at skin incision also reported significantly less pain during movement 24 h postoperatively. We interpret this association as a more effective regional anaesthesia in these patients.

NOL changes after skin incision and their association with postoperative pain may suggest that NOL may be used to evaluate block effectiveness during general anaesthesia. Bollag et al. also reported a difference in NOL response to skin incision in patients undergoing thoracoscopy with and without epidural anaesthesia [17] and concluded that regional anaesthesia effectiveness can be evaluated using NOL. We can confirm these findings with peripheral nerve blocks. Further, it can be stated that NOL response to a painful stimulus has an effect on pain levels in the early postoperative period. In a recent study on cardiac surgery patients, Balan et al. could show an opioid-sparing effect, both in the intraoperative and postoperative period in NOL-guided anaesthesia, with and without erector spinae plane block, as part of an ERAS protocol. In the control group without block, intraoperative NOL values were reported to be higher than in the block group [18]. Our results are in line with Balan et al., although there is no control group without regional anaesthesia, which may contribute to the fact that we did not observe a difference in postoperative opioid requirements. In addition, in our patients, absolute values of NOL were not associated with outcome parameters (postoperative pain and opioid consumption). The same was observed by Ledowski et al., reporting that the NOL response to skin incision rather than intraoperative NOL values predicts postoperative pain [19]. However, Ledowski’s patients were more heterogeneous and apparently did not receive regional anaesthesia. Additionally, we did not find a relationship between time-weighted averages in NOL and postoperative pain levels, which is in contrast to Ledowski’s report. We could only find a tendency for NOL TWA <10, which is somewhat surprising since this is outside the recommendations and would indicate an ideal nerve block or overdosing of opioids or both. While these results do not necessarily support an NOL-guided anaesthesia regime based on the proposed NOL corridor between 10 and 25, Meijer et al. showed a reduction in intraoperative opioids with an NOL-guided vs. a standard approach, which was associated with fewer hypotensive events [20]. No difference in early postoperative pain or opioid consumption was reported. The optimal target NOL during anaesthesia, especially with the use of regional anaesthesia, is still unclear. For their ERAS regime, Balan et al. used the regular NOL corridor from 10 to 25, as recommended by the manufacturer, to define optimal nociception–antinociception balance. It is possible that in our patients, intraoperative opioids were actually overdosed, which is again in line with results from Meijer et al. and which can be associated with intraoperative hypotension. Therefore, the evaluation of nerve block effectiveness is important to avoid an intraoperative overdose of opioids and conversely avoid insufficient analgesia in the case of block failure. The effectiveness of regional anaesthesia seems to be best evaluated by NOL changes after a noxious stimulus such as a skin incision. An NOL-guided approach may, nonetheless, be valuable with the use of regional anaesthesia, as suggested by previous studies [18,20]. However, the effect of an NOL-guided anaesthesia regime involving regional anaesthesia in orthopaedic joint surgery remains to be evaluated in future studies.

Naturally, there are some limitations inherent in this study. First, this is an observational study with relatively few cases, and the results should be interpreted in this context. Furthermore, we included both hip and knee arthroplasty surgery, and there may be differences in the NOL response to regional anaesthesia, which has to be considered when interpreting our results. Since not only patients with primary arthroplasty were included, the results may not be generally applicable in the setting of orthopaedic surgery. Finally, while our results propose the utility of NOL in the setting of joint arthroplasty, this may not apply to other surgical fields, although previous works suggest otherwise.

## 5. Conclusions

In our cohort of patients undergoing hip or knee arthroplasty, our data suggest that intraoperative nociception measurement can be used for the evaluation of peripheral nerve block effectiveness. We found a difference in NOL change after skin incision between groups reporting postoperative NRS > 3 vs. NRS ≤ 3 during movement on the first postoperative day, but not on the second, although a tendency could be observed.

In summary, it can be concluded that intraoperatively, nociception may be used to evaluate peripheral nerve blocks and could possibly have its value as an additional method for optimizing anaesthesia. These results remain to be confirmed in a larger cohort.

## Figures and Tables

**Table 1:** Demographic and surgery-related data. BMI: Body Mass Index, d1: first postoperative day (24 h after surgery), d2: second postoperative day (48 h after surgery), NRS: Numeric Rating Scale.

Parameter	Mean (SD)/ Median [Range]
Age (years)	68.6 (13.7)
Height (m)	172.3 (10.2)
Weight (kg)	85.7 (19.0)
BMI (kg/m^2^)	28.9 (6.1)
Duration anaesthesia (min)	220.6 (56.1)
Duration surgery (min)	134.5 (91.4)
NRS d1-Rest	1 [0–7]
NRS d1-Movement	3 [0–8]
NRS d2-Rest	1 [0–8]
NRS d2-Movement	2 [0–8]

**Table 2:** Intraoperative data of patients with high (NRS 4–10) vs. low (NRS 0–3) pain levels on the first postoperative day in movement. BIS: bispectral index, d1: first postoperative day (24 h after surgery), d2: second postoperative day (48 h after surgery), HR: heart rate, NOL: Nociception level index, NRS: Numeric Rating Scale (0–10), TWA: time-weighted average.

	NRS 0–3 (Median (Quartiles)) (*n* = 21)	NRS 4–10 (Median (Quartiles)) (*n* = 14)	*p*-Value
NOL post knife to skin	4.5 (3/24.8)	16 (3/29)	0.376
NOL reaction to knife to skin (pre vs. Post [%])	−12.3 (−54.6/53.6)	119 (51.1/262.5)	0.005
NOL during surgery (median)	7 (4/15)	5.5 (3/12)	0.268
HR post knife to skin	64 (54/71)	54 (48.2/62.8)	0.113
HR reaction to knife to skin (pre vs. Post [%])	0 (0/1.8)	0 (−1.5/1.2)	0.368
BIS post knife to skin	43.2 (38.8/51)	44.1 (39.6/44.5)	0.533
BIS reaction to knife to skin (pre vs. Post [%])	0 (−4.8/3.2)	1.5 (−0.3/2.7)	0.682
NOL TWA > 25	0.32 (0.2/0.6)	0.23 (0.2/0.3)	0.127
NOL TWA > 30	0.2 (0.1/0.4)	0.16 (0.1/0.2)	0.162
NOL TWA > 35	0.13 (0.1/0.3)	0.09 (0.1/0.1)	0.162
NOL TWA > 40	0.08 (0/0.2)	0.06 (0/0.1)	0.175
NOL TWA > 45	0.04 (0/0.1)	0.03 (0/0)	0.175
NOL TWA < 10	0.61 (0.4/0.8)	0.77 (0.7/1)	0.055
Surgery type	8 vs. 13 (knee vs. hip)	9 vs. 5 (knee vs. hip)	0.24

## Data Availability

The data presented in this study are not publicly available due to data protection concerns for sensitive data but are available on request and approval of the corresponding author.

## Supplementary Materials

The following supporting information can be downloaded at: https://www.mdpi.com/article/10.3390/medicina59061166/s1, Table S1: Intraoperative data of patients with high (NRS 4–10) vs. low (NRS 0–3) pain levels on the second postoperative day in movement. Figure S1: Associations between NOL values and morphine equivalents.
