# Supplementary material for: Influence of Intraoperative Nociception during Hip or Knee Arthroplasty with Supplementary Regional Anaesthesia on Postoperative Pain and Opioid Consumption
Source: Medicina (Kaunas). 2023 Jun 17;59(6):1166. doi: 10.3390/medicina59061166 (PMC10300707; doi:10.3390/medicina59061166)

Table S1: Intraoperative data of patients with high (NRS 4-10) vs. low (NRS 0-3) pain levels on the second postoperative day in movement. BIS: bispectral index, HR: heart rate, NOL: Nociception level index, NRS: numeric rating scale (0-10), TWA: time weighted average

	**NRS 0-3 (median (quartiles)) (*n*=22)**	**NRS 4-10 (median (quartiles)) (*n*=12)**	**P-value**
NOL post knife to skin	12.75 (3/28.2)	6.5 (3.2/23.8)	0.634
NOL reaction to knife to skin (pre vs. Post [%])	-11.1 (-40.9/100)	100 (58.3/150)	0.072
NOL during surgery (median)	24 (16/42)	20 (17.5/36.5)	0.662
HR post knife to skin	63.25 (49.5/71.8)	60.25 (53.8/64.2)	0.438
HR reaction to knife to skin (pre vs. Post [%])	0 (0/1.7)	0 (0/2.8)	0.768
BIS post knife to skin	44.5 (42.1/51)	38.9 (35.6/42.9)	0.018
BIS reaction to knife to skin (pre vs. Post [%])	1.5 (-0.3/3.2)	-0.3 (-12.6/2.7)	0.155
NOL TWA > 25	0.35 (0.3/0.5)	0.19 (0.2/0.3)	0.08
NOL TWA > 30	0.22 (0.2/0.4)	0.13 (0.1/0.2)	0.106
NOL TWA > 35	0.14 (0.1/0.2)	0.09 (0.1/0.1)	0.138
NOL TWA > 40	0.08 (0/0.1)	0.06 (0/0.1)	0.192
NOL TWA > 45	0.05 (0/0.1)	0.03 (0/0)	0.261
NOL TWA < 10	0.61 (0.5/0.7)	0.85 (0.6/1)	0.178
Surgery type	12 vs. 10 (knee vs. hip)	5 vs. 7 (knee vs. hip)	0.7

Figure S1: Associations between NOL values and morphine equivalents. Regression coefficients, 95% confidence intervals and P-values were obtained from univariable linear regression models. Response variables included morphine equivalents within the first day, second day and within two days after surgery. NOL: Nociception level index

NOL post knife to skin	Coefficient	CI	P-value
day 1	-0.16	-0.51 – 0.19	0.359
day 2	-0.37	-1.17 – 0.43	0.356
day 3	-0.53	-1.63 – 0.58	0.337
**NOL during surgery (median)**			
day 1	-0.57	-1.34 – 0.20	0.141
day 2	-1.08	-2.89 – 0.74	0.237
day 3	-1.65	-4.13 – 0.83	0.185

NOL reaction to knife to skin (pre vs. post [%])	Coefficient	CI	P-value
day 1	0.004	-0.01 – 0.02	0.441
day 2	0.01	-0.01 – 0.04	0.337
day 3	0.02	-0.02 – 0.05	0.352

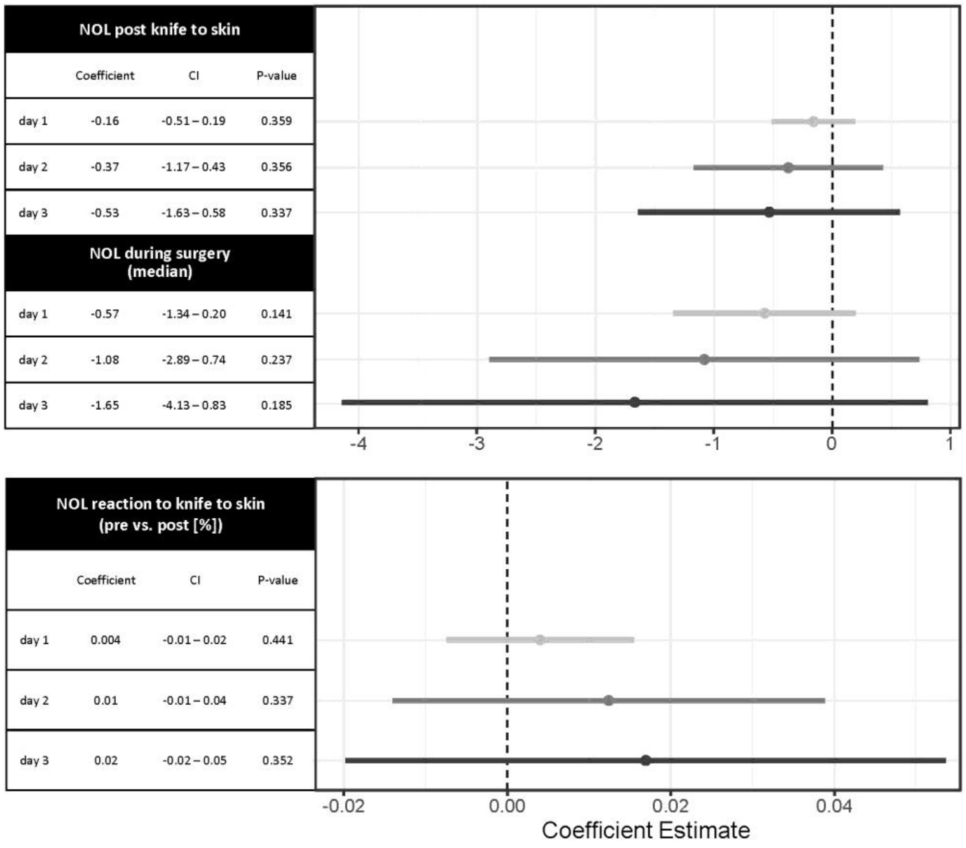

Supplement: Supplementary file 1 [file medicina-59-01166-s001.zip › medicina-2404221-supplementary.pdf]
